# Reliable structural interpretation of small-angle scattering data from bio-molecules in solution - the importance of quality control and a standard reporting framework

**DOI:** 10.1186/1472-6807-12-9

**Published:** 2012-05-17

**Authors:** David A Jacques, Jules Mitchell Guss, Jill Trewhella

**Affiliations:** 1 School of Molecular Bioscience Building, University of Sydney, G08 Butlin Avenue, Sydney, NSW, 2006, Australia

**Keywords:** Small-angle scattering, Protein structure, Reporting framework, Standards, Quality control

## Abstract

Small-angle scattering is becoming an increasingly popular tool for the study of bio-molecular structures in solution. The large number of publications with 3D-structural models generated from small-angle solution scattering data has led to a growing consensus for the need to establish a standard reporting framework for their publication. The International Union of Crystallography recently established a set of guidelines for the necessary information required for the publication of such structural models. Here we describe the rationale for these guidelines and the importance of standardising the way in which small-angle scattering data from bio-molecules and associated structural interpretations are reported.

## Background

The theory underpinning small-angle scattering (SAS) and its application to the study of polymer structures in solution have been known for over 80 years [1]. Early in the development of SAS methodology it was recognised that biomolecules were ideal candidates for structural characterisation because they can be prepared as a solution of identical, mono-disperse particles as a result of the fact that a given polypeptide or polynucleotide of defined sequence folds to form a well-defined structure [2]. However, until the last two decades only a few, readily prepared systems were studied by SAS. The modern ‘explosion’ in the use of the technique for structural biology was made possible by the advent of molecular biology and modern biochemistry tools for milligram-scale purification of proteins and polynucleotides for sample preparation; improvements in performance and availability of SAS instrumentation; and modern computing with easy-to-use software for data processing and interpretation. Perhaps the most attractive development to the structural biologist was the ability to generate coordinate files representing 3D structures, whether they be *ab initio* molecular envelope (bead) reconstructions, or atomic models based on the refinement of rigid body domains obtained by another method (*e.g.* crystallography, NMR, or high confidence homology models based on experimental structures) [3]. While such algorithms can generate visually appealing graphics, the inherently low information content of a 1D small-angle scattering profile from unoriented macromolecules in solution means that without careful evaluation of sample and data quality, as well as a good understanding the information limits of the data, the structures obtained can be misleading or simply wrong.

For the results of biomolecular-SAS studies to be relied upon by the community, a standard reporting framework is needed specifying what details of the experiment and its interpretation must be provided. A similar step was critical to the maturing of crystallography and NMR methods as they became automated and more available to the non-specialist. The International Union of Crystallography (IUCr) has acted to introduce a set of guidelines for such a standard reporting framework for the publication of biological SAS experiments. Here we expand upon the benefits of adopting this framework.

## Discussion

The phenomenon that gives rise to SAS is the same that produces diffraction from a crystal. The key difference between SAS and crystallography is the nature of the sample. In bio-molecular SAS, the sample usually consists of a macromolecule dissolved in an aqueous buffer, whereas crystallography relies on the molecules being aligned in three dimensions within the crystal. During the SAS experiment, the macromolecule is able to sample all possible orientations, and consequently the data represent a rotational average. This averaging results in a loss of information relative to diffraction data. The attraction of SAS is that, compared with crystallography, the experiments are quicker; the samples are in solution and may be measured over a range of conditions (pH, ionic strength, temperature, etc.); and there is no requirement for crystallinity, thereby reducing sample preparation time, and expanding the range of samples and conditions that may be amenable to structural characterisation.

However, a key yet often underappreciated difference between biological SAS and crystallographic samples is the importance of characterising sample quality. A ‘poor quality’ protein crystal yields no measureable diffraction. At this point data interpretation ceases - there are no data to process, so it is not possible to refine a model. In the event that a crystal does diffract, the quality of the data can be estimated from the resolution to which statistically significant diffraction can be observed and from the self-consistency of the data, as indicated by the averaging of equivalent reflections. In other words, crystallography has natural quality control checkpoints, as well as established reporting requirements and as a result, the coordinate files produced from a diffraction experiment carry a certain authority. In the case of a ‘poor quality’ SAS sample, data are still observed and can be measured so long as there is a macromolecule present that has a different scattering density to its supporting solvent. A poor quality sample would be one that fails the tests of containg a mono-disperse solution of non-interacting particles; a stringent requirement for accurate structural interpretation. The scattering data by themselves do not provide all the necessary evidence for sample quality. Independent characterization of sample properties are required; *e.g.* purity checks, concentration determination, and comparison with standards [4]. Without a set of adequate quality control checkpoints, SAS data can be processed and interpreted and incorrect models proposed. Consequently, coordinate files produced from SAS carry very little authority on their own. Without a community agreed reporting framework that requires the reporting of the quality control measures and the necessary information for independent evaluation, the correct structural data and models will have less impact than they deserve based on the very well-understood theory and principles of SAS.

As mentioned above, crystal structures are treated in the wider biological community as carrying an implicit correctness - though this is not strictly true. Atomic coordinates themselves are meaningless without the reporting of the appropriate data processing and refinement statistics. The convention of reporting these statistics in ‘Table 1’ of any crystallographic publication arose from the need of reviewers and the wider readership to be able to independently assess the conclusions that the authors draw from a given structure. This convention in crystallography was established through the intervention of the IUCr.

Due to the importance of demonstrating sample and data quality, the publication of SAS experiments for structural biology purposes requires a similar rigorous reporting framework. In this period wherein the application of SAS in structural biology has ‘blossomed’ it perhaps has been too easy to report SAS results with insufficient rigor. SAS, as an allied technique to crystallography, is benefitting from the experience and authority of the IUCr. At its 2011 congress, the IUCr’s Journals Commission adopted a set of guidelines for the publication of biological SAS data that had been prepared and agreed by the IUCr Small-Angle Scattering Commission. These guidelines are available at http://journals.iucr.org/services/sas/, and have been described in detail [5].

It should be stressed that the IUCr’s guidelines aim to establish a convention for those experiments that report structures in the form of atomic or bead coordinates. While the guidelines do not explicitly mention other types of experiment that may be performed by SAS (*e.g.* determining oligomeric equilibria, measuring natively unstructured proteins, etc.) [6] many of the recommended quality control measures will still be applicable (such as establishing the absence of non-specific aggregation, and the calculation of molecular mass for the scattering particles). It also should be stressed that the aim of these guidelines is not to define a level of quality that needs to be achieved in a SAS experiment, but rather to establish what information needs to be reported so that readers (including reviewers) are able to independently assess the interpretation and conclusions drawn from the data by the authors.

## Conclusions

SAS instruments at synchrotrons and neutron sources are among the most heavily subscribed at these facilities. This demand has led to the construction of new SAS instruments at facilities the world over. The availability of these instruments and the continuing development of easy to use software for analysis is expanding the SAS community beyond dedicated SAS ‘specialists’. Consequently, the importance of implementing a standard publication framework for SAS structural biology has never been greater. As such the initiative of the IUCr in leading the establishment of such a framework is most welcome and we would recommend that the framework be broadly adopted.

## Abbreviations

SAS, Small-angle scattering; IUCr, International Union of Crystallography.

## Competing interests

The authors declare that they have no competing interests.

## Author’s contributions

DAJ, JMG, and JT all contributed to the drafting and critical revision of the manuscript. All authors read and approved the final manuscript.
